# Molecular Docking Studies and Anti-Tyrosinase Activity of Thai Mango Seed Kernel Extract

**DOI:** 10.3390/molecules14010257

**Published:** 2009-01-07

**Authors:** Saruth Nithitanakool, Pimolpan Pithayanukul, Rapepol Bavovada, Patchreenart Saparpakorn

**Affiliations:** 1 Department of Pharmacy, Faculty of Pharmacy, Mahidol University, Bangkok 10400, Thailand; saruth_pipe@yahoo.com (S. N.); 2 Department of Pharmaceutical Botany, Faculty of Pharmaceutical Sciences, Chulalongkorn University, Bangkok 10300, Thailand; E-mail: brapepol2@hotmail.com (R. B.); 3 Department of Chemistry, Faculty of Science, Kasetsart University, Bangkok 10900, Thailand; E-mail: patchareenart_s@yahoo.com (P. S.)

**Keywords:** *Mangifera indica* L, Molecular docking study, Polyphenols, Pentagalloylglucopyranose, Tyrosinase inhibitor.

## Abstract

The alcoholic extract from seed kernels of Thai mango (*Mangifera indica* L. cv. ‘Fahlun’) (Anacardiaceae) and its major phenolic principle (pentagalloylglucopyranose) exhibited potent, dose-dependent inhibitory effects on tyrosinase with respect to L-DOPA. Molecular docking studies revealed that the binding orientations of the phenolic principles were in the tyrosinase binding pocket and their orientations were located in the hydrophobic binding pocket surrounding the binuclear copper active site. The results indicated a possible mechanism for their anti-tyrosinase activity which may involve an ability to chelate the copper atoms which are required for the catalytic activity of tyrosinase.

## Introduction

Tyrosinase (E.C. 1.14.18.1) is a multifunctional Cu-containing enzyme which is widely distributed in nature. The enzyme is mainly involved in the first two steps of melanin biosynthesis, which consists of the hydroxylation of L-tyrosine (monophenolase activity) and the oxidation of the product of this reaction, the L-DOPA (diphenolase activity), to the corresponding *o*-quinone [1]. Tyrosinase, which is contained in vegetables, fruits and mushrooms, is a key enzyme in the browning which occurs upon brushing or long term storage [2]. In mammals, this enzyme is responsible for skin pigmentation abnormalities such as flecks and defects [3]. Tyrosinase is also linked to Parkinson’s and other neurodegenerative diseases [4, 5], oxidizing excess dopamine to produce DOPA quinones, highly reactive compounds which induce neuronal damage and cell death. 

The crystallographic structure of tyrosinase has been established recently [6], enabling a close look at its three-dimensional structure and a better understanding of its mechanism of action. This three-dimensional structure revealed the presence of a hydrophobic protein pocket adjoining the binuclear copper active site. Each of these sites, CuA and CuB, are coordinated by three nitrogen donor atoms from histidine residues: CuA binds to the ε-nitrogen atoms of His^38^, His^54^, and His^63^, and CuB binds to those of His^190^, His^194^, and His^216^.

As plants are a rich source of bioactive chemicals, which are mostly free from harmful side-effects, there is an increased interest to identify natural tyrosinase inhibitors from plants. Tannins are diverse compounds with a wide variation in structure and concentration within and among plant species. Therefore, biomedical research on the health benefits of these compounds is of great interest. Mangos (*Mangifera indica* L.), which belong to the family *Anacardiaceae*, grow in tropical and subtropical regions, and their components are commonly used in folk medicine to produce a wide variety of remedies [7, 8, 9]. Among the edible portions, the mango seed kernel has been shown to exhibit potent antioxidant activity and to have a relatively high phenolic content [10]. The total antioxidant capacity and the amount of polyphenolic constituents present vary considerably from one variety of plant to another [11, 12]. The ethanolic extract of Thai mango seed kernel (MSKE) cultivar ‘Fahlun’ was found to contain 61.28% pentagalloylglucopyranose (PGG), 0.68% methyl gallate (MG) and 0.44% gallic acid (GA); the MSKE and its isolates exhibited potent free radical scavenging, antioxidant and anti-inflammatory activity as well as anti-hepatotoxicity against liver damage induced by carbon tetrachloride in rats [13]. In this study, the MSKE and its isolated phenolic principles were evaluated for their inhibitory effects on L-DOPA oxidation by mushroom tyrosinase. We also performed molecular docking studies using the Gold v3.2 program with the aim of explaining the differences in activity of the plant polyphenols isolated from MSKE. Understanding how the tannin principles in the extract interact with tyrosinase may explain how they inhibit tyrosinase and melanin formation.

## Results and Discussion

### Effect of MSKE and its phenolic principles on the activity of mushroom tyrosinase

**Figure 1:**
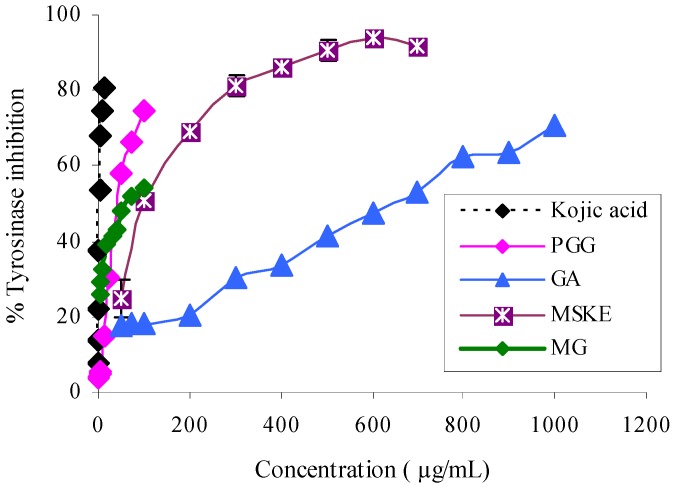
Dose-dependent inhibition of mushroom tyrosinase by MSKE, GA, MG, PGG and the positive reference (KA). Tyrosinase activity was measured using L-DOPA as the substrate. Each value represents mean ± SEM (n = 2).

Figure 1 shows the dose-response curves of MSKE and its phenolic principles (GA, MG and PGG) and a well-known tyrosinase inhibitor, kojic acid (KA) on the inhibition of L-DOPA oxidation by mushroom tyrosinase. It can be seen that MSKE and its phenolic principles clearly showed a concentration-dependent inhibitory activity against tyrosinase. The order of potency as judged by the half-inhibition concentration (IC_50_) was KA (2.21 ± 0.05 µg/mL) > PGG (42.65 ± 1.85 µg/mL) > MG (62.50 ± 0.50 µg/mL) > MSKE (98.63 ± 1.62 µg/mL) > GA (644.00± 14.00 µg/mL) (Table 1). These results indicated that the anti-tyrosinase potency of MSKE may be attributed to its major principle (PGG) and other unidentified constituents, since PGG exerted its effect at the lowest IC_50_ value and had the highest percentage content (61.28%) within the MSKE compared with GA and MG. Although the anti-tyrosinase potency of MG was close to PGG, its percentage content within the MSKE was very low (0.68%), therefore MG may only have a negligible effect, similar to GA (0.44%). The results for MSKE were in accordance with that of Kim *et al*. [14] who found that *Galla rhois* which contains PGG as its major constituent, exhibited potent anti-tyrosinase activity. Since MSKE, GA, MG and PGG have been demonstrated to possess chelating activity when compared to EDTA [13]; a possible mechanism for their anti-tyrosinase activity may involve the chelation of copper atoms which are required for the catalytic activity of tyrosinase [14, 15]. Therefore, we performed docking calculations to explain the possible mechanism of the phenolic principles isolated from MSKE towards the binding site of tyrosinase.

**Table 1:** Tyrosinase inhibitory activity of MSKE and its phenolic principles compared with the positive reference, KA.

Test compounds	Anti-tyrosinase
	IC_50_ ± SEM (µg/mL)
MSKE	98.63 ± 1.62
Phenolic principles of MSKE [13]:	
GA	644.00± 14.00
MG	62.50 ± 0.50
PGG	42.65 ± 1.85
Positive reference: KA	2.21 ± 0.05

### Molecular docking study

**Figure 2:**
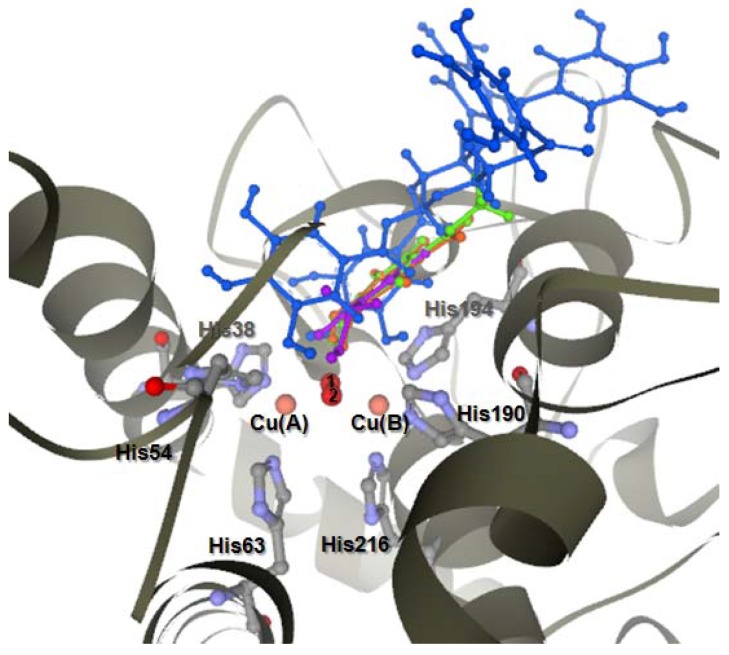
Docked conformation of ligand structures in the binding site of tyrosinase. KA (purple), PGG (blue), MG (green), GA-ionize (orange).

The selected docked conformations of KA, GA, MG and PGG in the tyrosinase binding site are shown in Figure 2. The docked conformations revealed that all ligands were located in the hydrophobic binding pocket surrounding the binuclear copper active site. The location of the docked compounds agreed well with that of docked 2,4-resorcinol derivatives which would contribute to their tyrosinase inhibitory potency [16]. 

**Figure 3:**
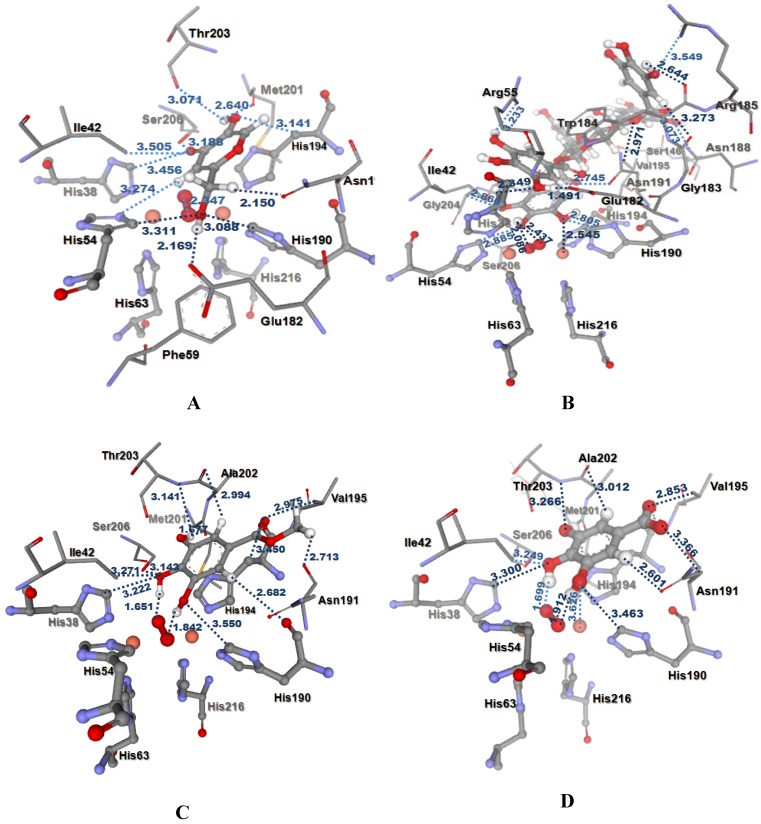
Distances (in Å) between residues in the tyrosinase binding pocket and the ligands: (A) KA, (B) PGG, (C) MG, (D) ionized GA.

In this study, all docked ligands were found to have some interaction between an oxygen atom of the ligands and CuB within 4 Å. Moreover, these docked conformations also formed an H-bonding interaction (<3.00 Å) with peroxide in the active site. In the binding pocket, common H-bonding interactions were formed between all docked ligands and Ile42, Asn191, Thr203 and Ser206. The specific H-bonding interaction with Phe59 was only found in the docked conformation of KA. In order to explain the binding of these compounds, the H-bonding interactions with the other surrounding residues in the hydrophobic binding pocket were also investigated. In Figure 3A, strong H-bonding interactions between the hydroxyl group of KA and an oxygen atom of Glu182 and Met201 were formed. H-bonding interactions were also formed with His38, His54, His190, and His194 which are important residues coordinated with the two copper ions in the active site. In addition, a π-π interaction between KA and His194 was found. The docked PGG, MG, and GA are shown in Figure 3B-D. Additional similar H-bonding interactions with His38, His190, Val195, and Ala202 and a π-π interaction with His194 were found in their docked conformations. In the case of docked PGG, H-bonds with Arg55, Ser146, Val147, Glu182, Gly183, Trp184, Arg185, Asn188, and Gly204 were also formed with PGG but these H-bonding interactions were not found with MG and GA. However, a strong H-bond with Met201 was found in the docked MG and GA. Docked MG and GA revealed similar binding modes with the exception of the methyl group in the MG structure. The methyl group in the MG structure showed an extra H-bond with an oxygen atom on the backbone Asn191. The docking results agreed well with the observed *in vitro* data, which showed that the tyrosinase inhibitory activity of PGG was higher than those of MG and GA, respectively. 

## Conclusions

MSKE and its major phenolic principle (PGG) possess potent, dose-dependent anti-tyrosinase activity with respect to L-DOPA. The docking results agreed well with the observed *in vitro* data, in which the anti-tyrosinase activity of its phenolic principle PGG was higher than those of the other phenolic principles MG and GA, respectively. The docking study revealed the binding orientation of the phenolic principles in the tyrosinase hydrophobic binding pocket surrounding the binuclear copper active site, which resulted in inhibition of enzyme activity.

## Experimental

### Chemicals

Mushroom tyrosinase (E.C. 1.14.18.1) and L-3,4-dihydroxyphenylalanine (L-DOPA; ≥ 98%) were obtained from Sigma Chemical Co. (St. Louis, MO, USA). Gallic acid (GA; ≥ 98%), kojic acid (KA; ≥ 98%) and methyl gallate (MG; ≥ 98%) were purchased from Fluka (Buchs, Switzerland). Pentagalloylglucopyranose (PGG; > 95%) was obtained from Endotherm GmbH (Germany). All reagents used in the *in vitro* experiment were obtained from commercial sources and were of analytical grade.

### Plant materials

Fully grown unripened Thai mango fruits (*Mangifera indica* L. cv. ‘Fahlun’) (Anacardiaceae) were purchased from a local market. The voucher specimen (RB 20007) was deposited at the Herbarium of the Faculty of Pharmaceutical Sciences, Chulalongkorn University, Thailand. Fresh seeds were homogenized in a blender, using hot ethanol (80°C). After removal of the solvent under reduced pressure, the extract was defatted with hexane, evaporated, and then freeze-dried to afford a crude mango seed kernel extract (MSKE) with a yield of 8.66% (w/w).

### Standardization

GA, MG and PGG were used as chemical markers and the calibration curves for each compound were obtained by densitometric scanning of different quantities of the chemical marker bands on developed chromatographic plates. An aliquot of the crude extract (MSKE) (8 μL, 25 mg/mL) was applied along with serial amounts of the chemical marker stock solution. The thin-layer chromatographic (TLC) plates were developed in a pre-saturated twin trough glass tank using CHCl_3_/MeOH/EtOAc/ethyl methyl ketone (6:1.6:2:2) with five drops of formic acid as the mobile phase for GA and MG and CHCl_3_/EtOH/formic acid (3:5:1) for PGG. The developed TLC plates were scanned at 286 nm and the amount of each compound (GA 4.4, MG 6.8 and PGG 612.8 mg/g dry weight) in MSKE was calculated from the calibration curves [13].

### Determination of mushroom tyrosinase inhibition *in vitro*

The dopachrome method described by Iida *et al*. [17] was followed with slight modifications. Briefly, 20 mM phosphate buffer (pH 6.8, 120 µL), 48 U/ml mushroom tyrosinase (40 µL) and various sample concentrations (20 µL) with or without enzyme were placed in the wells of a 96-well microplate. After pre-incubation at 25°C for 10 min, 0.85 mM L-DOPA (20 µL) was added and subsequently incubated at 25°C for 20 min. The amount of dopachrome was measured at 492 nm in an ELISA reader (Anthos 2010, Wals, Austria) and KA was used as a positive tyrosinase inhibitor control. The extent of inhibition by the test samples was expressed as the percentage of concentration necessary to achieve 50% inhibition (IC_50_). 

### Molecular modeling

**Figure 4:**
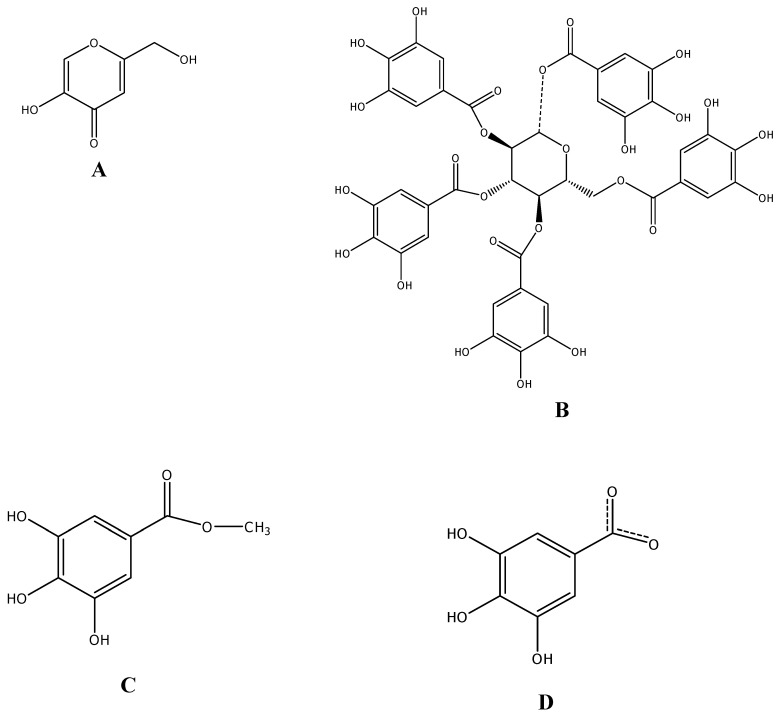
Structures of the studied compounds. (A) kojic acid, (B) 1,2,3,4,6-penta-O-galloyl-β-D-glucopyranose, (C) methyl gallate and (D) ionized gallic acid.

Docking studies of PGG, MG and GA and the positive reference (KA) were performed. The structures of these compounds (Figure 4) were constructed and optimized at the HF/3-21G level of theory using a Gaussian 03 program [18]. To prepare the tyrosinase structure, the crystal structure of the oxy form of tyrosinase was taken from the Protein Data Bank (PDB code 1wx2) [6]. The caddie protein (ORF378) and water molecules were removed. Hydrogen atoms were added to the enzyme using the SYBYL version 7.2 program (TRIPOS Assoc., Inc., St. Louis, MO, USA). The molecular docking method was performed using the Gold version 3.2 program [19] to study the binding orientation of PGG, MG, GA (ionize) and KA into the tyrosinase structure. The radius of the binding site was set to 10 Å. The default parameters of the automatic settings were used to set the genetic algorithm parameters. The docked conformation which had the highest GoldScore was selected to analyze the mode of binding.

### Statistical analysis

The sample concentration which provided 50% inhibition (IC_50_) was calculated from the graph which plotted inhibition percentage against sample concentration. The data were expressed as mean ± standard error of the mean (SEM). All statistical analyses were carried out using Minitab^®^ Release 14 for Windows (Minitab Inc., State College, PA). Analysis of variance was performed by ANOVA procedures. Significant differences between the means were determined by Tukey’s pairwise comparison test at a level of *P* < 0.05.
